# Sentiments Evoked by WHO Public Health Posts During the COVID-19 Pandemic: A Neural Network-Based Machine Learning Analysis

**DOI:** 10.7759/cureus.19141

**Published:** 2021-10-30

**Authors:** Tanmay S Pathak, Harsh Athavale, Amey S Pathak, Sunita Athavale

**Affiliations:** 1 Electronics and Communication Engineering, International Institute of Information Technology Hyderabad (IIITH), Hyderabad, IND; 2 Computer Science and Engineering, Manipal University Jaipur, Jaipur, IND; 3 Medicine and Surgery, Rajasthan University of Health Sciences (RUHS), Jaipur, IND; 4 Anatomy, All India Institute of Medical Sciences Bhopal, Bhopal, IND

**Keywords:** social media analytics, bert, who- world health organization, covid 19, neural networks, sentiment analysis

## Abstract

Introduction

The World Health Organization (WHO) is a specialized agency of the United Nations responsible for international public health. Established on April 7, 1948, it has since played a pivotal role in several public health achievements and has had considerable success. But never since the establishment of the WHO has it faced a pandemic of such a huge scale. The spread of the coronavirus and the inability of the WHO to contain it has raised many questions about its efficiency and role. The present study explores the range of emotions and sentiments evoked by public health information posts of WHO over the course of the pandemic.

Methods

This study uses Bidirectional Encoder Representations from Transformers (BERT), which is a neural network-based technique for natural language processing. Three timeframes of five months each, starting from March 2020, were defined. A total of six posts, two posts from each timeframe, were then analysed. Comments were classified as positive, neutral and negative. The broader positive and negative classes were further subclassified into two classes each. Natural language processing was further applied to obtain results.

Results

The general trend of the sentiments over the period of pandemic showed a significant and dominant proportion of negative comments that overshadowed the neutral, positive and irrelevant comments over all timeframes. Specifically, the negative sentiments peaked during the second timeframe. The negativity was directed more towards the WHO, governments and people not complying with coronavirus disease 2019-appropriate norms. Positive comments were mostly expressed towards health workers.

Conclusion

An unusually high proportion of negative sentiment was observed in response to relatively innocuous public health posts. This may be a result of heightened anxiety, questionable credibility of the sources of information and geopolitical power play maligning the image of the WHO.

## Introduction

The World Health Organization (WHO) is mandated with ensuring the highest possible level of health globally [1]. Since its establishment in 1948, the WHO has been at the forefront of improving public health by pushing for universal healthcare, monitoring public health risks, coordinating responses to health emergencies, and promoting human health and well-being [2]. The past has shown that the WHO has played a central role, leading to significant advancements in the public health sphere, most notably being the eradication of smallpox and the near eradication of polio [3,4]. But in 2019, with the onset of coronavirus disease 2019 (COVID-19), the WHO, for the first time, faced a pandemic of such a huge magnitude and global span. Spanning 220 countries and territories and with over 200 million reported cases (as of October 2021), it has been the biggest public health crisis since the inception of the WHO [5]. The emergence of the COVID-19 pandemic in 2020 necessitated a global response spearheaded by the WHO. The WHO outlines its global response in leading the fight against the COVID-19 pandemic by helping countries prepare and respond, providing accurate information and bust dangerous myths, ensuring that vital supplies reach health line workers, training and mobilizing healthcare workers and aiding the search for a vaccine [6].

With the pandemic already labelled as an infodemic, providing accurate and useful guidance to the general public had been deemed essential [7]. Tagliabue et al. while referring this pandemic as the pandemic of dis-information, concluded that the extensive reach of mass media and social networks has unfortunately contributed to fake reports, myths and confusion [8]. The role of the WHO also came under intense scrutiny, with Brown and Ladwig highlighting many media reports that were critical towards the WHO’s role in handling the COVID-19 pandemic, which raised questions on the efficacy and need of the WHO [9]. This distrust in the WHO led to psychological distress in the general population. Few studies have analysed these prevailing sentiments and emotions portrayed by the general public during the pandemic. Khan et al. conducted a survey on emotions specifically analysing anger among the general population and concluded that anger contributed to the spread of misinformation as the angry individuals tend to consider even the false claims as credible [10].

The present study explores the range of emotions and sentiments evoked by public health information posts posted by WHO in the best interest of the public as per its mandate. The present study also aims to gain an insight into whether the information overload coupled with the questions over the role of WHO prompted an unusual response to such innocuous public health information posts.

## Materials and methods

Dataset

In order to analyse the sentiments of the general population evoked by communications from the WHO, we chose to analyse Facebook posts from the official WHO Facebook page. This allowed us to analyse sentiments of the general population towards actions, announcements and awareness campaigns by the WHO aimed at managing the COVID-19 pandemic.

We first defined three timeframes (T1, T2 and T3) of five months each, starting from March 2020. We choose a total of six posts, with two posts from each timeframe. The posts were identified based on their content and engagement. The posts that addressed the primary concerns of the public in that particular timeframe like apprehension about general COVID-19 protocol, adapting to the new normal and concern regarding vaccines were chosen. The chosen posts had at least 1800 comments to ensure a tenable prediction model and to have a general sense of prevailing sentiments.

T1 Timeframe, March 2020 to July 2020

Post 1 (P1) consisted of a cartoon depicting a man sneezing into his elbow in public transport. The post also advocated for the above-mentioned action. None of the characters in the cartoon had worn a mask [11]. Post 2 (P2) consisted of suggestions to healthcare workers on stress-coping strategies during the pandemic [12].

T2 Timeframe, August 2020 to December 2020

Post 3 (P3) attempted to bring back a feeling of normalcy by indicating that the COVID-19 virus is here to stay and called the period “new normal” [13]. Post 4 (P4) suggested guidelines to avoid the 3Cs: Crowded places, Close contact sitting and Confined settings during the period of “new normal” [14].

T3 Timeframe, January 2021 to May 2021

Post 5 (P5) depicted an animation asserting the role of COVAX in managing COVID-19. Through the post, the WHO urged that even with the introduction of the vaccines, “no one is safe till everyone is safe” [15].​​​​​​​ Post 6 (P6) attempted to squash rumors pertaining to the safety and efficacy of the vaccine [16].

Labelling

A total of 3000 comments were selected randomly from the scraped posts and were used for hand labelling, which were then used for training and testing the neural network. The labelling was collaboratively done with the consensus of all the authors to reduce bias.

The data was labelled into five categories that encompassed a broad range of positive and negative emotions associated with the comments. The labels were as follows: A, angry; B, concerned; C, neutral; D, agree; E, laudatory.

Hateful and angry comments were labelled as A; negatively sarcastic comments and comments displaying emotions of concern and forlorn were labelled as B. Labels D and E were associated with positive comments, with comments of agreement being labelled as D and laudatory comments showing emotions of thankfulness labelled as E. Comments that included personal advice and irrelevant stats along with comments that had no remote connection to the post were labelled as C.

Preprocessing

The scraped data is highly unstructured and requires some amount of preprocessing before the corpus is utilized for training. Preprocessing includes removing special symbols, removing duplicate comments, converting the whole corpus to lowercase, removing stopwords and removing comments that are less than or equal to three characters. Furthermore, the cleaned dataset was tokenized using the Bidirectional Encoder Representations from Transformers (BERT) tokenizer.

Training and testing

Out of the 3000 labelled comments, 80% were used for training and 20% for testing. The BERT_BASE_ model was used, which is a pre-trained model that helps computers to deconstruct complex sentences posted in the comments section by extracting contextual meaning and then further classifies them into different labels. We then fine-tuned the pre-trained model to work specifically on our classification problem. Finally, the same trained neural network was used to predict the labels of 4420 comments from the T1 timeframe, 5256 comments from the T2 timeframe and 3902 comments from the T3 timeframe.

## Results

The BERT-based neural network classified the entire dataset with an overall accuracy of 85.27%. Table 1 shows the f1 scores achieved for each label.

**Table 1:** Scores achieved by each label

Label	f1 score
A	0.72
B	0.30
C	0.45
D	0.05
E	0.70

Analysis of sentiments over the three timeframes is as follows.

Timeframe 1

The health education posts by the WHO after the initial outbreak concerned with the prevention of infection, and coping with stress. The sentiment was overwhelmingly negative and was characterized by anger and concern. The neutral/irrelevant comments were slightly lesser in comparison. The share of positive sentiments, which consisted of agreement, laudation and thankfulness, was disproportionately low as compared to the proportion of negative sentiment (Table 2). Within negative sentiments, anger dominated over the milder forms of negative sentiments such as concern, fear and sarcasm (Table 3). Stronger negative sentiments were primarily directed towards the WHO for not providing pertinent advice and also towards the general public not compliant with COVID-19-appropriate behavior.

**Table 2:** Percentage of posts classified into three labels

Timeframe	Percentage of comments classified under each sentiment label (%)			Percent total (%)
	Negative	Neutral	Positive	
T1	49.3	38.4	12.3	100.0
T2	74.9	23.2	1.9	100.0
T3	62.5	33.1	4.4	100.0
T1, T2, T3	63.0	31.0	6.0	100.0

**Table 3:** Percentage of posts classified into five labels

Timeframe	Percentage of comments classified under each sentiment label (%)					Percent total (%)
	A (Anger)	B (Concern)	C (Neutral)	D (Agree)	E (Laudatory)	
T1	28.9	20.4	38.4	3.3	9.0	100.0
T2	42.1	32.8	23.2	1.0	0.9	100.0
T3	36.6	25.8	33.1	2.4	2.0	100.0

On the other hand, though the positive sentiments were in minority, laudatory and thankfulness sentiments predominated over milder positive sentiments of agreeability (Table 3). The positive laudatory sentiment was predominantly directed towards the untiring efforts of the health workers.

Timeframe 2

The next phase of the pandemic generally contained posts concerning the resumption of routine activities while advocating for appropriate precautionary measures. The results showed an overwhelming feeling of “too little too late”, which was predominated by anger directed towards the WHO and an overall dissatisfaction towards the effectiveness of the WHO in managing the pandemic.

Among all the three timeframes, we observed that negative sentiments peaked during this timeframe. Within negative sentiments, anger once again dominated over concern. On the other hand, the overall positivity appreciably reduced in this timeframe. Furthermore, we also noticed a sharp drop in the percentage of posts classified as neutral (Tables 2, 3).

Timeframe 3

The posts in the third timeframe were characterized by dispelling the concerns regarding vaccines and ensuring vaccinations for all. It was observed that the overall negative sentiment continued to persist and the proportion of negative sentiments in the T3 timeframe was only slightly less than in the T2 timeframe. Anger, once again, was the dominant negative sentiment. On the other hand, while the proportion of positive sentiments grew slightly, it was still very low as compared to the proportion of negative sentiments (Tables 2, 3).

The negative sentiments were not limited to the WHO but were also directed towards respective governments and their federal health bodies. These sentiments were not limited to institutions but were also aimed at wealthy and influential personalities.

**Figure 1:**
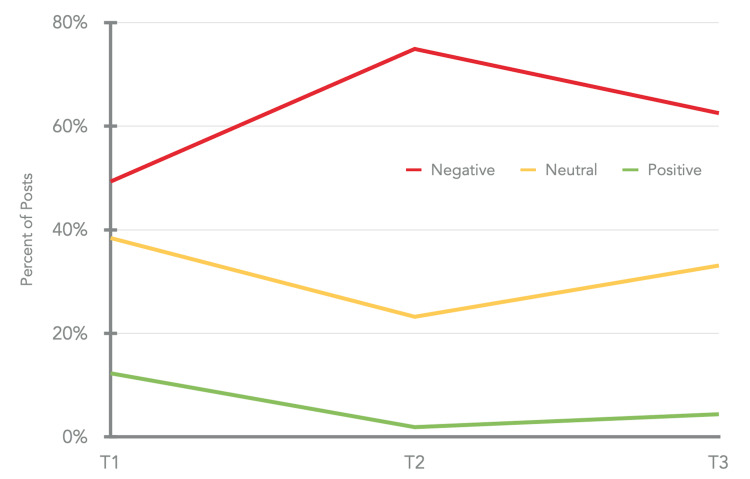
Evolution of sentiments during the pandemic

The general trend of sentiments over all the three timeframes of pandemic combined showed that just 6% of all assessed comments corresponded to positive comments, 31% of all the comments corresponded to neutral sentiment while a massive 63% of all comments corresponded to the negative sentiment with negative comments overshadowing the neutral, positive and irrelevant comments in all three timeframes. The negativity represented in comments peaked in the T2 timeframe. Furthermore, even though the negativity reduced slightly in the T3 timeframe, it did not convert into positive sentiments (Table 2, Figure 1).

## Discussion

An unprecedented health crisis like the COVID-19 pandemic affected not just the physical but also the emotional and psychological well-being of society at large, thereby also affecting the economic well-being of the general population [17]. Lwin et al. observed that among the general population, the negative sentiments shifted from fear to anger during the course of the pandemic [18]. Aslam et al. observed that even the news headlines of several global English language news stations evoked mainly negative sentiments of fear, sadness and anger [17]. Similar to these observations, the present study reaffirms the dominance of negative sentiments during the pandemic. The negative sentiments were dominated by anger that further accentuated with the course of the pandemic. The positivity in people’s sentiments was appreciably low and was restricted towards public health workers and gratitude for their service, as was observed by Lwin et al. as well [18]. This finding was in line with the findings of the present study, with positive sentiments being minimal throughout the pandemic.

An international platform like the WHO, entrusted with coordinating responses to health emergencies, is expected to play a pivotal role in providing accurate, timely and evidence-based credible information for public consumption to educate, alleviate fear and anxiety and dispel misinformation. The WHO made several provisions for this by putting up various public health posts. It also added a messaging-based (WhatsApp) service for spreading awareness and addressing fake news [19]. However, it was observed in the present study that the negativity reflected in response to public health posts by the WHO was to a considerable extent targeted towards the perceived complacency, incompetence and even complicity of governments in dealing with the pandemic. According to Kuznetsova, though the role of the WHO as a source for information and knowledge dissemination was severely criticised, this was due to the uncertain and rapidly evolving situation and the lack of data and knowledge about the new virus and the disease [20]. Emeziem, in an excellent review on COVID-19 pandemic and global health policy, has emphatically underscored the enormous contribution WHO has made as an international organisation towards global health. According to Emeziem, the controversy entangled with the WHO was a result of strategic rivalries between its member states, i.e., US and China, to the extent of threatening its financing [1].

In a utopia, one can expect an ideal platform for evidence-based information like the official pages of WHO, but with the explosion of the use of social media, a situation far from this was created. Tagliabue et al. observed that the mass media did not play its desired role and rather spread misinformation and the social media networks contributed to the spread of misinformation and denial of the scientific literature. This, according to them, resulted in the appearance of rather harmful attitudes [8]. Bernard et al. emphasised that many conspiracy theories have been circulating misinformation regarding the origin, spread, cure and vaccination of COVID-19, which were even endorsed by political leaders, with very little evidence backing these theories. It has been observed to affect the sentiments of people, especially when endorsed by influential people or political leaders that tends to have severe negative downstream effects. Vaccine hesitancy is one such example that has severely compromised the fight against COVID-19 [21]. Lwin et al. advocated that negative emotions need to be monitored and countered with strategic public health communication [18]. Han et al. in their survey conducted on emotions infer that angry individuals contribute to the spread of misinformation and tend to consider even the false claims to be scientifically credible, which might lead to vaccine hesitancy that severely compromises the fight against COVID-19 [22].

Kuznetsova states that the pandemic underscored the need and importance of an international organisation like the WHO as controlling pandemics is not possible without international cooperation due to their transboundary nature. The WHO is the only source of legally binding international regulations for the pandemic response, and hence, the establishment of its authority and integrity is paramount [20]. According to Emeziem, it is important not to destroy the institutions for fulfilling national interests. Emeziem also recommends that international organisations like the WHO ought to be insulated from political and global strategic games [1]. Additionally, Cowper states that people’s agreeability and involvement is contingent upon whether the communication is coming from experts or governments with an underlying political motive [23]. Kuznetsova recommends that the WHO should work on increasing its credibility with special attention to transparency, political and business neutrality and adapting evidence-based policy [20].

## Conclusions

The enormity of the health crisis, which led to isolation and economic crisis, coupled with questionable information sources on social media and aspersions on the credibility of the WHO created such a concoction that even the innocuous public health information posts by the WHO were misconstrued, generating overall negativity in the public psyche. This highlights that during such a crisis, it is very important to realise the importance of upholding the public morale, and concerted efforts have to be made to address both the psychological and emotional well-being of people.
